# Inclusion of unexposed clusters improves the precision of fixed effects analysis of stepped-wedge cluster randomized trials with binary and count outcomes

**DOI:** 10.1186/s12874-024-02379-z

**Published:** 2024-10-28

**Authors:** Kenneth Menglin Lee, Grace Meijuan Yang, Yin Bun Cheung

**Affiliations:** 1 Centre for Quantitative Medicine, Duke-NUS Medical School, Singapore, 169857 Singapore; 2 Division of Supportive and Palliative Care, National Cancer Centre Singapore, Singapore, 169610 Singapore; 3 Lien Centre for Palliative Care, Duke-NUS Medical School, Singapore, 169857 Singapore; 4 Signature Programme in Health Services & Systems Research, Duke-NUS Medical School, Singapore, 169857 Singapore; 5 Tampere Center for Child, Adolescent and Maternal Health Research, Tampere University, Tampere, 33520 Finland; 6 Center for Quantitative Medicine, Duke-NUS Medical School, 8 College Road, Singapore, 169857 Singapore

**Keywords:** Stepped wedge trials, Cluster randomized trials, Fixed effects model, Precision, Binary outcomes, Count outcomes, Generalized linear model

## Abstract

**Background:**

The fixed effects model is a useful alternative to the mixed effects model for analyzing stepped-wedge cluster randomized trials (SW-CRTs). It controls for all time-invariant cluster-level confounders and has proper control of type I error when the number of clusters is small. While all clusters in a SW-CRT are typically designed to crossover from the control to receive the intervention, some trials can end with unexposed clusters (clusters that never receive the intervention), such as when a trial is terminated early due to safety concerns. It was previously unclear whether unexposed clusters would contribute to the estimation of the intervention effect in a fixed effects analysis. However, recent work has demonstrated that including an unexposed cluster can improve the precision of the intervention effect estimator in a fixed effects analysis of SW-CRTs with continuous outcomes. Still, SW-CRTs are commonly designed with binary outcomes and it is unknown if those previous results extend to SW-CRTs with non-continuous outcomes.

**Methods:**

In this article, we mathematically prove that the inclusion of unexposed clusters improves the precision of the fixed effects intervention effect estimator for SW-CRTs with binary and count outcomes. We then explore the benefits of including an unexposed cluster in simulated datasets with binary or count outcomes and a real palliative care data example with binary outcomes.

**Results:**

The simulations show that including unexposed clusters leads to tangible improvements in the precision, power, and root mean square error of the intervention effect estimator. The inclusion of the unexposed cluster in the SW-CRT of a novel palliative care intervention with binary outcomes yielded smaller standard errors and narrower 95% Wald Confidence Intervals.

**Conclusions:**

In this article, we demonstrate that the inclusion of unexposed clusters in the fixed effects analysis can lead to the improvements in precision, power, and RMSE of the fixed effects intervention effect estimator for SW-CRTs with binary or count outcomes.

**Supplementary Information:**

The online version contains supplementary material available at 10.1186/s12874-024-02379-z.

## Background

In a cluster randomized trial (CRT), clusters, instead of individuals, are randomized to receive the intervention. The stepped-wedge cluster randomized trial (SW-CRT) is an increasingly popular type of CRT where all clusters begin the trial unexposed to the intervention. Clusters are then randomized to begin implementing the intervention at different periods or “steps.” This crossover is uni-directional and is planned to continue until all clusters have been exposed to the intervention [1, 2].

Various statistical models have been suggested and applied for analyzing SW-CRT data [3]. Cross-sectional SW-CRTs are often analyzed using a “Hussey and Hughes” (H&H) mixed effects model [2, 3]. The H&H mixed effects model treats clusters as random and periods as fixed effects, producing a simple exchangeable within-cluster correlation structure [2]. In mixed effects models, the cluster random effect is uncorrelated with both the residual error term and other model covariates [4]. As a result, the mixed effects intervention effect estimator can become biased and inconsistent if the cluster random effect is correlated with other model covariates, such as in the presence of unmeasured cluster-level time-invariant confounders [4–8].

Furthermore, mixed effects models mainly rely on randomization to balance and control for known and unknown confounders. However, SW-CRTs often have fewer clusters than standard parallel CRTs, which can make balancing cluster characteristics difficult [9–11]. A 2016 review found that around 20% of published SW-CRTs had only 5 or fewer clusters [12]. A more recent 2023 review reported that the median number of cross-over sequences was 5, with the majority of studies randomizing 1 cluster to each sequence [13]. Moreover, when the number of clusters is small, modelling clusters as random effects in a H&H mixed effects model can result in inflated type I error rates and overly narrow confidence intervals for the intervention effect estimates [14–16]. Notably, modelling clusters as fixed effects in a fixed effects model do not exhibit this inflated type I error rate when the number of clusters is small, over a variety of different degrees of freedom [14].

Fixed effects models are often used to control for all cluster-level time-invariant confounders [5, 6, 8]. Some previous studies have elected to use a fixed effects model to analyze data collected from a SW-CRT, citing difficulties that arise from having a small number of clusters [17–19], such as concerns of cluster-level confounders [17]. The fixed effects model has also been used in a trial with a stepped-wedge design that had practical and logistical issues preventing randomization [20].

When analyzing a SW-CRT with a fixed effects model, the clusters serve as the fixed effects units of interest. Fixed effects analyses for longitudinal studies of individuals are often referred to as only making “within-[unit] comparisons” [5] where “only covariates that vary within-[units] at the observational level should be used in the model” [6], and “cases that do not change either (1) do not contribute much information to the analysis or (2) are altogether omitted by design” [21]. Some authors have even explicitly stated that “comparisons are made within [units] rather than between [units]… Thus, only those who have experienced both the outcome and the exposure of interest are included” [22].

In principle, all clusters in a SW-CRT spend some periods unexposed and exposed to the intervention. This within-cluster variation in intervention status allows for within-cluster comparisons to help estimate the intervention effect. However, in practice, some clusters may end the trial unexposed to the intervention due to early termination of the trial [23] or by design [24, 25]. We will discuss a motivating case study that also concluded with an unexposed cluster due to the restructuring of hospital management. Under the previously described phrasing and guidance regarding fixed effects analyses, one may have the impression that an unexposed cluster in a SW-CRT would not meaningfully contribute to the fixed effects intervention effect estimate and may be excluded from the analysis.

Fortunately, it has been recently proven that including an unexposed cluster in a fixed effects analysis of SW-CRTs with continuous outcomes will improve the precision of the intervention effect estimator [26]. However, more than half of the published SW-CRT articles covered in a 2016 review had binary outcomes [12], and it was not clear in the previous work by Lee et al. [26] if the same conclusion could be generalized to non-continuous outcomes. Famously, Robinson and Jewell showed that the precision gain resulting from covariate-adjustment in regression models with continuous outcomes did not necessarily extend to logistic regression models [27]. Therefore, further work is required to explicitly demonstrate that including an unexposed cluster in a fixed effects analysis of SW-CRTs with non-continuous outcomes will improve the precision of the intervention effect estimator.

First, we will mathematically prove that the inclusion of unexposed clusters also increases the precision of the intervention effect estimator in a fixed effects model for SW-CRTs with binary and count outcomes. We then use simulations with binary and count outcomes to assess the impact of including unexposed clusters on the fixed effects intervention effect estimator in terms of precision, bias, coverage probability, power, type I error, and root mean square error. Additionally, we will re-analyze a SW-CRT of a novel palliative care model with binary outcomes and an unexposed cluster.

## Methods

### Analysis model and precision

**Fig. 1:**
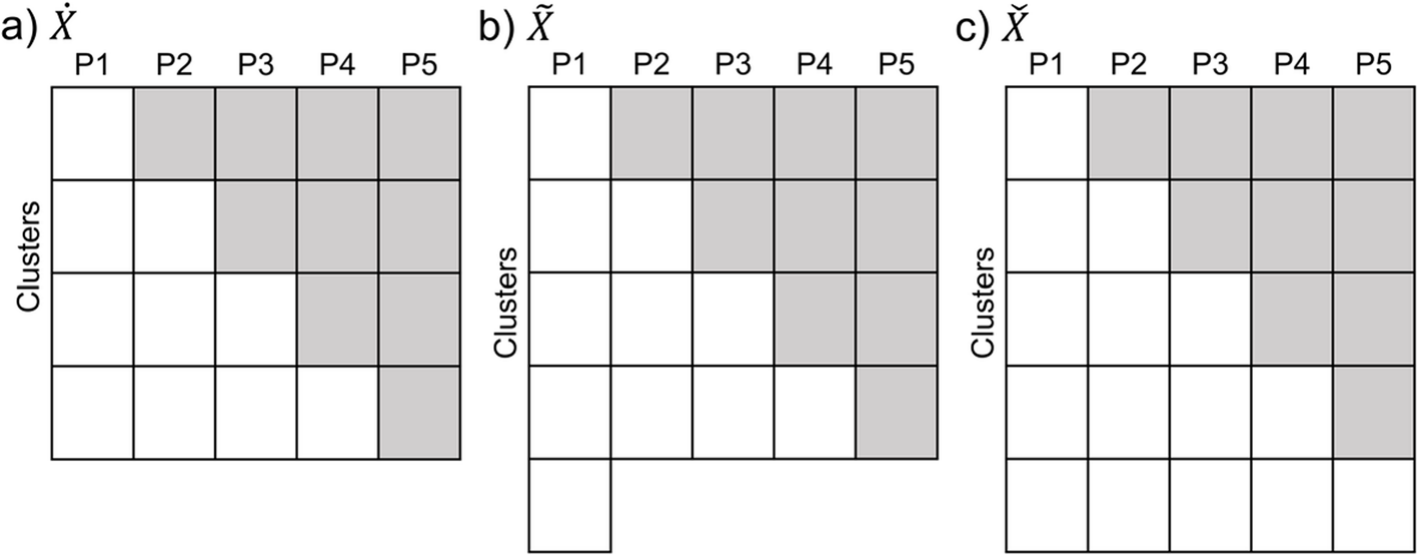
SW-CRT designs for $\dot{X}$, $\tilde X$, and $\check{X}$ are shown below. **a** $\dot{X}$ contains observations from only the exposed clusters. **b** $\tilde X$ contains observations from the exposed clusters and period 1 of the unexposed cluster. **c** $\check{X}$ contains observations from both the exposed and unexposed clusters

We begin with the 5 cluster, 5 period structure of the palliative care SW-CRT that motivated this research. The fifth cluster is never exposed to the intervention (thus serving as an unexposed cluster) (Fig. 1c).

Data collected from SW-CRTs with binary outcomes are analyzed using a fixed effects Logistic regression:

1 $$\:\text{l}\text{o}\text{g}\text{i}\text{t}\left(\text{E}\left[{Y}_{ijk}\right]\right)=\text{log}\left(\frac{{p}_{ijk}}{1-{p}_{ijk}}\right)=\delta{Z}_{ij}+{\phi}_{j}+{\alpha}_{i},$$

and for count responses, the fixed effects Poisson regression:

2 $$\:\text{l}\text{o}\text{g}\left(\text{E}\left[{Y}_{ijk}\right]\right)=\delta{Z}_{ij}+{\phi}_{j}+{\alpha}_{i}.$$

Here ${Y}_{ijk}$ is the binary or count health outcome for individual $k$ ($k=1,\dots,{n}_{i,j}$) in cluster $i$ ($i=1,\dots,5$) during period $j$ ($j=1,\dots,5$). $\delta$ is the constant intervention effect, $Z_{ij}$ is the intervention indicator (${Z}_{ij}=1$, if exposed to intervention, ${Z}_{ij}=0$ otherwise), ${\phi}_{j}$ are the fixed effects for each period (${\phi}_{1}=0$ for identifiability), and ${\alpha}_{i}$ are the fixed effects for each cluster.

We can re-write the fixed effects Logistic and Poisson regression as:

$$\text{l}\text{o}\text{g}\text{i}\text{t}\left(\text{E}\left[Y\right]\right)=X\beta$$

and:

$$\text{l}\text{o}\text{g}\left(\text{E}\left[Y\right]\right)=X\beta,$$

where $X$ is an $N\times10$ design matrix, with $N$ being the total number of study participants, and $\beta=(\delta,{\phi}_{2},..,{\phi}_{5},{\alpha}_{1},..,{\alpha}_{5})'$.

The variance-covariance matrix of the parameters is then:

$$\text{Var}\left(\widehat{\beta}\right)={\left({X}'VX\right)}^{-1},$$

where $V=diag\left(v\left(\mu\right)\right)$ and $v\left(\mu\right)$ is the $N$ by $1$ vector of all $v\left({\mu}_{ijk}\right)$. In logistic regression for binary outcomes:

$$v\left({\mu}_{ijk}\right)={\mu}_{ijk}\left(1-{\mu}_{ijk}\right)={p}_{ijk}\left(1-{p}_{ijk}\right)$$

where:

$${p}_{ijk}=\frac{{e}^{X\beta}}{1+{e}^{X\beta}}.$$

In Poisson regression for count outcomes:

$$v\left({\mu}_{ijk}\right)={\mu}_{ijk}={e}^{X\beta}.$$

Simply, $v\left(\mu\right)$ can be interpreted as a vector of non-negative weights, and $X'VX$ is a positive-definite matrix of the marginal weighted sum of observations in each parameter. For example, ${X}'VX{|}_{\text{1,1}}$ is the sum of weights $v\left({\mu}_{ijk}\right)$ for all exposed observations that receive the intervention.

We compare three different ways of using the data from a trial with an unexposed cluster (Fig. 1). Let (a) $\dot{X}$ be the $\dot{N}\times9$ design matrix containing observations from only the exposed clusters, (b) $\tilde{X}$ be the $\tilde{N}\times10$ design matrix for the analysis containing observations from the exposed clusters and period 1 of the unexposed fifth cluster, and (c) $\check{X}$ be the $\check{N}\times10$ design matrix for all clusters, including the unexposed cluster (Fig. 1). $\dot{N}$, $\tilde{N}$, and $\check{N}$ are the total sample sizes in the three models. Subsequently, $\dot{X}'\dot{V}\dot{X}$, $\tilde{X}'\tilde{V}\tilde{X}$, and $\check{X}'\check{V}\check{X}$ are $9\times9$, $10\times10$, and $10\times10$ product matrices explicitly defined in the Online Supplementary Material S1. Let $\dot{\delta}$, $\tilde{\delta}$, and $\check{\delta}$ be the GLM intervention effect estimators based on these three models.

The design matrix $\tilde{X}$ (Fig. 1b) serves two purposes in our proof. It helps demonstrate that unless the unexposed cluster contributes to the estimation of period effects, which requires observations from at least two periods, its inclusion will not improve the precision of the intervention effect estimate beyond analyzing only the exposed clusters, $\dot{X}$. Additionally, it facilitates the proof by providing a same-sized product matrix $\tilde{X}'\tilde{V}\tilde{X}$ for comparison with the product matrix $\check{X}'\check{V}\check{X}$ which includes all periods of the unexposed cluster.

### Proof of $\boldsymbol V\boldsymbol a\boldsymbol r(\dot\delta)=\boldsymbol V\boldsymbol a\boldsymbol r(\widetilde\delta)$

$\tilde{X}'\tilde{V}\tilde{X}$ contains observations from the exposed clusters and only period 1 of the unexposed cluster. Accordingly, the top left block matrix of the $9\times9$ elements in ${\tilde{X}}'\tilde{V}\tilde{X}$ is equal to $\dot{X}'\dot{V}\dot{X}$:

$$\widetilde X'\widetilde V\widetilde X=\left(\begin{array}{cc}\dot X'\dot V\dot X&0\\0&\sum\nolimits_kv\left(\mu_{\text{5,1},k}\right)\end{array}\right).$$

The inverted matrix ${\left({\tilde{X}}'\tilde{V}\tilde{X}\right)}^{-1}$ is then:

$$\left(\widetilde X'\widetilde V\widetilde X\right)^{-1}=\left(\begin{array}{cc}\left(\dot X'\dot V\dot X\right)^{-1}&0\\0&1/\sum\nolimits_kv\left(\mu_{\text{5,1},k}\right)\end{array}\right).$$

Therefore, $${\left(\tilde{X}'\tilde{V}\tilde{X}\right)}_{\text{1,1}}^{-1}={\left({\dot{X}}'\dot{V}\dot{X}\right)}_{\text{1,1}}^{-1}$$ and:

$$\text{V}\text{a}\text{r}\left(\tilde{\delta}\right)={\left(\tilde{X}'\tilde{V}\tilde{X}\right)}_{\text{1,1}}^{-1}={\left({\dot{X}}'\dot{V}\dot{X}\right)}_{\text{1,1}}^{-1}=\text{Var}\left(\dot{\delta}\right).$$

### Proof of $\text{V}\text{a}\text{r}\left(\check{\delta}\right)<\text{V}\text{a}\text{r}\left(\tilde{\delta}\right)$

For brevity, we present an abbreviated proof of $\text{V}\text{a}\text{r}\left(\check{\delta}\right)<\text{V}\text{a}\text{r}\left(\tilde{\delta}\right)$. The complete proof can be found in the Online Supplementary Material S2.

First, we represent ${\check{X}}'\check{V}\check{X}$ and $\tilde{X}'\tilde{V}\tilde{X}$ in terms of submatrices:

$${\check{X}}'\check{V}\check{X}=\left(\begin{array}{cc}\check{A}&\check{B}'\\\check{B}&\check{D}\end{array}\right),$$

$${\tilde{X}}'\tilde{V}\tilde{X}=\left(\begin{array}{cc}\tilde{A}&\tilde{B}'\\\tilde{B}&\tilde{D}\end{array}\right).$$

Submatrix $\check{A}=\tilde{A}$ is a scalar equal to the sum of the weights $v\left({\mu}_{ijk}\right)$ for all exposed participants. Submatrix $\check{B}=\tilde{B}$ is the 9 by 1 vector of marginal weighted sums for participants who receive the intervention in each period and cluster. Submatrices $\check{D}$ and $\tilde{D}$ represent the 9 by 9 weighted matrix product of the rows and columns of the design matrix that represent the dummy variables for the periods and clusters. Accordingly:

$$\text{V}\text{a}\text{r}\left(\check{\delta}\right)={\left({\check{X}}'\check{V}\check{X}\right)}_{\text{1,1}}^{-1}={(\check{A}-{\check{B}}'{\check{D}}^{-1}\check{B})}^{-1},$$

$$\text{V}\text{a}\text{r}\left(\tilde{\delta}\right)={\left({\tilde{X}}'\tilde{V}\tilde{X}\right)}_{\text{1,1}}^{-1}={(\tilde{A}-{\tilde{B}}'{\tilde{D}}^{-1}\tilde{B})}^{-1}.$$

Given that $\check{X}'\check{V}\check{X}$ and $\tilde{X}'\tilde{V}\tilde{X}$ are same-sized positive definite matrices ($\check{X}'\check{V}\check{X}\succ0$ and $\tilde{X}'\tilde{V}\tilde{X}\succ0$), then their principal submatrices $\check{D}$ and $\tilde{D}$ are also positive definite matrices ($\check{D}\succ0$ and $\tilde{D}\succ0$) [28]. The difference between $\check{D}$ and $\tilde{D}$ is positive semi-definite, where ${x}'\left(\check{D}-\tilde{D}\right)x\ge0$ for all $x$ in ${\mathbb{R}}^{9}$ [28]. Therefore, $\check{D}$ and $\tilde{D}$ can be ordered as induced by Loewner partial ordering [28]:

$$\check{D}\succcurlyeq\tilde{D},$$

and:

$${\check{D}}^{-1}\preccurlyeq{\tilde{D}}^{-1}.$$

Given that vector $\check{B}=\tilde{B}$(here on referred to as $\mathbb{B}$) and scalar $\check{A}=\tilde{A}$ (here on referred to as $\mathbb{A}$), then:

$${\left(\mathbb{A}-{\mathbb{B}}'{\check{D}}^{-1}\mathbb{B}\right)}^{-1}\le{\left(\mathbb{A}-{\mathbb{B}}'{\tilde{D}}^{-1}\mathbb{B}\right)}^{-1},$$

and:

$$\text{V}\text{a}\text{r}\left(\check{\delta}\right)\le\text{V}\text{a}\text{r}\left(\tilde{\delta}\right).$$

In Online Supplementary Material S2, we additionally prove by contradiction that $\text{V}\text{a}\text{r}\left(\check{\delta}\right)\ne\text{V}\text{a}\text{r}\left(\tilde{\delta}\right)$.

Altogether, we demonstrate that $\text{V}\text{a}\text{r}\left(\check{\delta}\right)<\text{V}\text{a}\text{r}\left(\dot{\delta}\right)$, and the inclusion of an unexposed cluster improves the precision of the fixed effects intervention effect estimator in GLMs with binary or count outcomes. Furthermore, including an always-exposed cluster (a cluster that never receives the control condition) is equivalent to including an unexposed cluster in terms of reducing the variance of the intervention effect estimator (Online Supplementary Material S3). These results are easily generalizable to any number of exposed and unexposed clusters.

### Simulation

In this section, we simulate datasets with binary and Poisson count outcomes over a variety of scenarios to assess the impact of including unexposed clusters on the fixed effects intervention effect estimator in terms of precision, bias, coverage probability, power, type I error, and root mean square error.

### Simulation settings

We simulated SW-CRT data with binary and count outcomes based on a generalized linear model with an exchangeable within-cluster correlation structure:

$$g\left(\text{E}\left[{Y}_{ijk}\right]\right)=\delta{Z}_{ij}+{\phi}_{j}+{\alpha}_{i}$$

for individual $k$ ($k=1,\dots,{n}_{i,j}$) in cluster $i$ ($i=1,\dots,I$) during period $j$ ($j=1,\dots,J$). $\delta$ is the constant intervention effect, ${Z}_{ij}$ is the intervention indicator (${Z}_{ij}=1$, if exposed to intervention, ${Z}_{ij}=0$ otherwise), and ${\phi}_{j}$ are the fixed effects for each period (${\phi}_{1}=0$ for identifiability). Unlike Eqs. 1 and 2, here ${\alpha}_{i}\sim\left({\mu}_{\alpha},{{\tau}}_{{\alpha}}^{2}\right)$ is specified as a random effect for the ${i}^{\text{th}}$ cluster.

We used a logit and log-link function for the binary and count outcomes, respectively. With binary health outcomes, $E\left[{Y}_{ijk}\right]$ was the probability of an event ${p}_{ijk}$. With count health outcomes, $E\left[{Y}_{ijk}\right]$ was the Poisson mean ${\mu}_{ijk}$.

### Simulation of binary outcomes

For binary outcomes, we generated the true intervention effects $\delta$ equal to $0$ and $\text{l}\text{n}\left(1.25\right)$. We set the period effect equal to an linear increase of $0.1$ per period. To simulate cluster effects, we set the ${\mu}_{\alpha}$ to $\text{ln}\,\left(0.30/0.70\right)$ and variance ${\tau}_{\alpha}^{2}$ to $0.0332$, $0.1731$, and $0.3655$, to generate corresponding ICC values of $0.01$, $0.05$, and $0.1$, where the ICC is approximated as $\rho=\frac{{\tau}_{\alpha}^{2}}{{\tau}_{\alpha}^{2}+\frac{{\pi}^{2}}{3}}$ [29].

### Simulation of Poisson count outcomes

For count outcomes, we generated the true intervention effects $\delta$ equal to $0$ and $\text{l}\text{n}\left(0.80\right)$. We set the linear period effect equal to an increase of $0.1$ per period. To simulate cluster effects, we set the between-cluster average ${\mu}_{\alpha}$ to $\text{l}\text{n}\;\left(0.30\right)$ and variance ${\tau}_{\alpha}^{2}$ to $0.015$, $0.077$, and $0.16$, to generate corresponding ICC values of $0.01$, $0.05$, and $0.1$ where the ICC is approximated as $\rho=\frac{{\tau}_{\alpha}^{2}}{{\tau}_{\alpha}^{2}+\text{l}\text{n}(1+\frac{1}{{e}^{{\mu}_{\alpha}}})}$ [30].

### Simulation scenarios

**Fig. 2:**
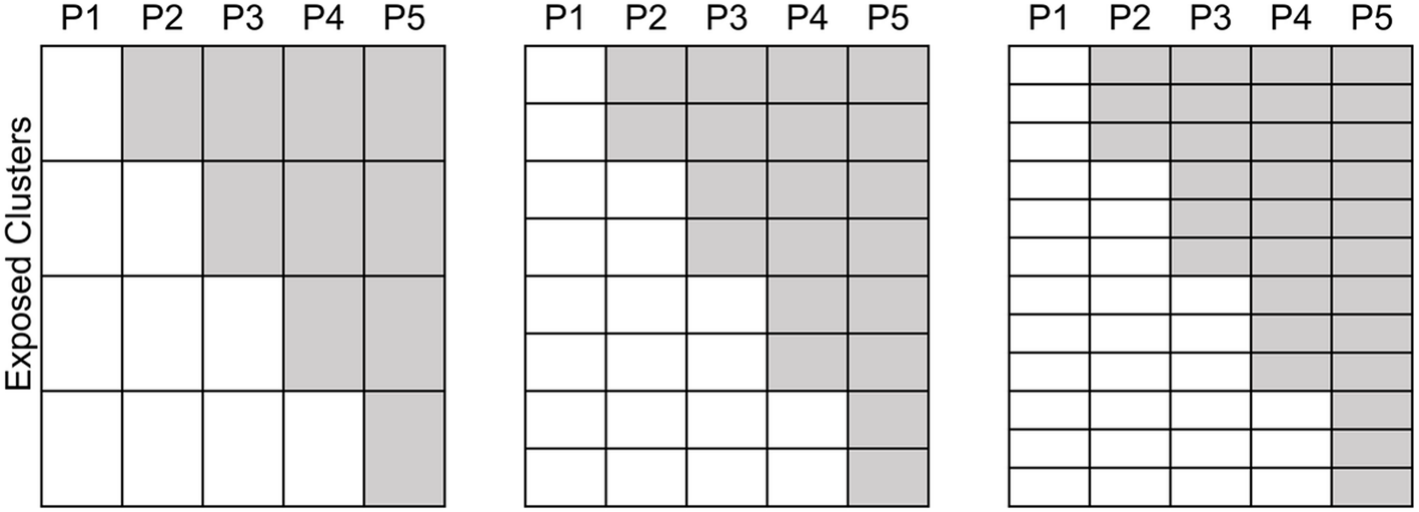
Exposed clusters in the simulation of SW-CRT designs with 4, 8, or 12 exposed clusters across 5 periods (4 steps). Each of these designs is then simulated with an additional 0, 1, 2, 3, or 4 unexposed clusters

We simulated scenarios with 4, 8, or 12 exposed clusters and 0, 1, 2, 3, or 4 unexposed clusters, altogether producing $I=$ 4 + 0, 4 + 1, 4 + 2, 4 + 3, 4 + 4, 8 + 0, …, or 12 + 4 total clusters. All scenarios had a fixed total of $J=5$ periods (4 steps) with either 1, 2, 3, or 4 clusters crossing from control to intervention at each step, depending on the number of exposed clusters (4, 8, or 12) (Fig. 2).

Each scenario had a range of different average cluster sample sizes across the $$J$$ periods of the $${i}^{th}$$ cluster, where $${n}_{i}\sim\left(k,\theta\right)$$ with $$k=$$$$30$$, $$100$$, and $$300$$, and $$\theta=1$$, producing an average cluster sample size $$\text{E}\left[{n}_{i}\right]$$ of $$30$$, $$100$$ or $$300$$ individuals. Realized sample sizes for cluster $$i$$ and period $$j$$ were subsequently generated with $$n_{i,j}\sim Poisson\left(n_i\right)$$.

In total, 270 scenarios were investigated (3 # of exposed clusters $\times$ 5 # of unexposed clusters $\times$ 2 intervention effects $\times\:3\:\text{v}\text{a}\text{l}\text{u}\text{e}\text{s}\:\text{o}\text{f}\:{\tau}_{\alpha}^{2}\times3\:\text{c}\text{l}\text{u}\text{s}\text{t}\text{e}\text{r}\:\text{s}\text{a}\text{m}\text{p}\text{l}\text{e}\:\text{s}\text{i}\text{z}\text{e}\text{s}$) for both binary and count outcomes.

### Analysis of simulated data

We generated $s=2000$ simulated datasets for each simulation scenario and estimated the intervention effect ${\widehat{\delta}}_{s}$ using the corresponding fixed effects models (Eqs. 1 & 2). In our results, we present the absolute bias $(\text{A}\text{b}\text{s}\:\text{B}\text{i}\text{a}\text{s}=[{\sum}_{s=1}^{2000}{\widehat{\delta}}_{s}/2000]-\delta)$ when $\delta=0$, and the relative bias $(\text{R}\text{e}\text{l}\:\text{B}\text{i}\text{a}\text{s}=[\text{A}\text{b}\text{s}\text{o}\text{l}\text{u}\text{t}\text{e}\:\text{b}\text{i}\text{a}\text{s}/\delta]\times100)$ when $\delta\ne0$. Precision is the reciprocal of the average variance estimates $(\text{P}\text{r}\text{e}\text{c}\text{i}\text{s}\text{i}\text{o}\text{n}=1/\left[{\sum}_{s=1}^{2000}{\text{V}\text{a}\text{r}(\widehat{\delta}}_{s})/2000\right])$. We provide the type I error rate when $\delta=0$ and the power when $\delta\ne0$ for rejecting the null hypothesis of $\delta\le0$ for binary outcomes and $\delta\ge0$ for count outcomes at the one-sided significance level of 0.05. CP is the probability that the 95% confidence interval contains the true effect. RMSE is the square root of the average squared difference between the estimated effect ${\widehat{\delta}}_{s}$ and the true effect $\delta$ over the 2000 simulated data sets for each scenario $\left(\text{R}\text{M}\text{S}\text{E}=\sqrt{{{\sum}_{s=1}^{2000}{[\widehat{\delta}}_{s}-\delta]}^{2}/2000}\right)$. The Monte Carlo standard errors (standard deviation of the 2000 estimated intervention effects ${\widehat{\delta}}_{s}$ for each scenario) are included in the Online Supplementary Material S6.

## Results

### Simulation results

**Fig. 3:**
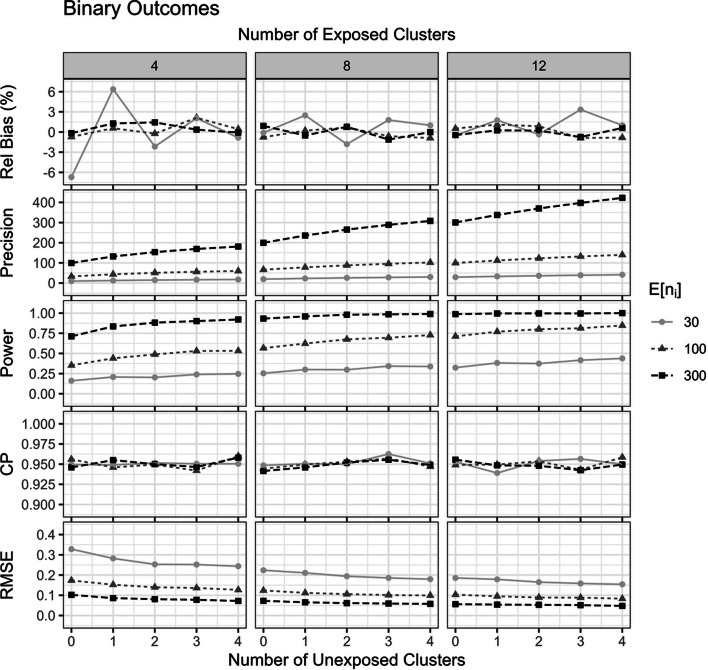
The impact of including unexposed clusters on properties of the fixed effects intervention effect estimator for binary outcomes. Results are presented across number of exposed clusters, unexposed clusters, average cluster size $E\left[n_i\right]$, with fixed true intervention effect $\delta=\text{log}\,\left(1.25\right)$ and $ICC=\left(0.05\right)$

**Fig. 4:**
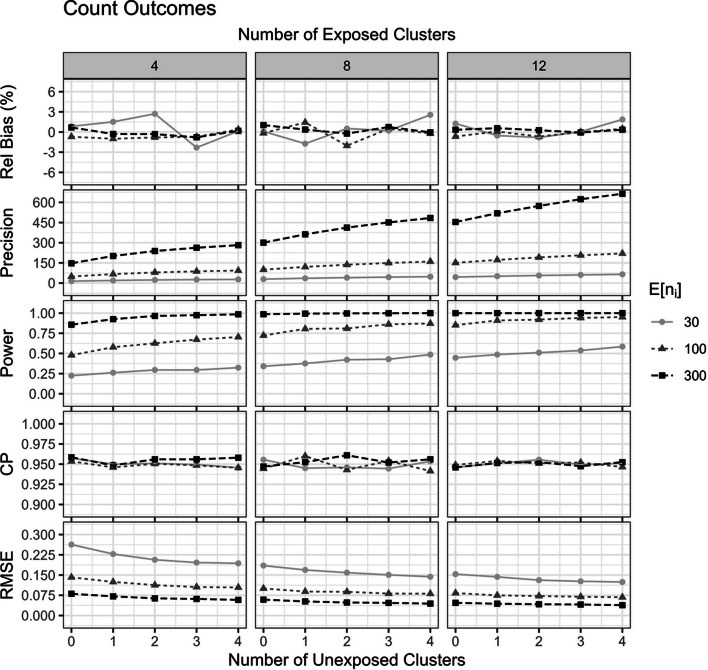
The impact of including unexposed clusters on properties of the fixed effects intervention effect estimator for count outcomes. Results are presented across number of exposed clusters, unexposed clusters, average cluster size $E\left[n_i\right]$, with fixed true intervention effect $\delta=\text{log}\,\left(0.80\right)$ and $ICC=\left(0.05\right)$

Figures 3 and 4 show the simulation results for binary outcomes with true intervention effect $\delta=\text{l}\text{n}\,\left(1.25\right)$ and Poisson count outcomes with true intervention effect $\delta=\text{l}\text{n}\,\left(0.80\right)$, respectively, in scenarios with an ICC of $0.05$. Overall, the fixed effects analyses yielded practically unbiased intervention effect estimates with CP of 95% confidence interval close to the nominal level (Figs. 3 and 4). The inclusion of unexposed clusters had no impact on these two properties.

The inclusion of unexposed clusters increased the precision, power, and reduced the RMSE of the fixed effects intervention effect estimator for both binary and count outcomes (Figs. 3 and 4). The more unexposed clusters included, the better the improvement. In both the fixed effects logistic and Poisson regression, the precision gain of including 4 unexposed clusters was roughly equal to the precision gain of randomizing an additional 4 clusters to receive the intervention (Figs. 3 and 4).

The results from other scenarios are qualitatively similar to those reported here for both binary and count outcomes and are included in the Online Supplementary Material S4 and S5. Scenarios when $\delta=0$ all maintained near 5% type I error rates, regardless of the inclusion of unexposed clusters (Online Supplementary Material S4 and S5).

### Case study results

In this section, we re-analyzed data collected from a SW-CRT of a novel palliative care co-rounding model (intervention) against a standard palliative care delivery model (control) for cancer inpatients [31]. In the standard care model, oncologists conducted the daily ward rounds as usual and referred patients to the palliative care department when they considered it appropriate. In the novel co-rounding model, oncologists and palliative care specialists conducted the ward rounds together and initiated palliative care as per their joint consensus.

For the purposes of this re-analysis, we only kept the first admission if a patient was admitted more than once during the study period ranging from December 2017 to July 2019. In total, there were 3462 hospital first admissions. The primary outcome for this trial was hospital length of stay, treated as a continuous variable. In this article, we will focus on the binary secondary outcomes: (i) hospital readmission within 30 days following the first admission and (ii) use of specialist palliative care.

The study was initially designed as a standard 4 cluster SW-CRT with 5 (4 month long) periods, with clusters defined as different oncology teams in the Singapore General Hospital. However, after restructuring of hospital management, an additional fifth oncology team was developed and deployed after the original 4 clusters had already been randomized and just before study initiation. Given that the fifth cluster was not included in the randomization process, it implemented the standard care model for the entire duration of the trial, effectively serving as an unexposed cluster.

The original analysis of the SW-CRT [31] used a fixed effects model to account for cluster effects, control for cluster-level confounders, and address concerns over the robustness of applying the mixed effects model to a SW-CRT with such a small number of clusters [14, 15]. The unexposed cluster was not included in the original analysis as the implications of including an unexposed cluster in a fixed effects model were not methodologically clear at the time.

Here, we re-analyze the data using the fixed effects model, with and without the unexposed cluster. Including an unexposed cluster would only be sensible if its underlying period effects matched that of the exposed clusters [11, 32]. Currently, there are no standard practices for evaluating this assumption. In this case, we estimated the Akaike Information Criterion (AIC) and Bayesian Information Criterion (BIC) of the models excluding and including interaction terms between the unexposed cluster and period indicators for readmission outcomes (AIC: 3781.908 and 3782.068, BIC: 3843.404 and 3868.162, respectively) and use of specialist palliative care outcomes (AIC: 3291.124 and 3297.671, BIC: 3352.62 and 3383.766, respectively). Both AIC and BIC favored the models that excluded the interaction terms, assuming same period effects between the exposed and unexposed clusters.

We estimated the intervention effects for (i) hospital readmission within 30 days (Table 1) and (ii) use of specialist palliative care (Table 2) among all cancer patients and among only stage III/IV cancer patients. The stage III/IV cancer patients were expected to be the primary beneficiaries of palliative care. In general, including the unexposed clusters in the fixed effects Logistic regression models yielded smaller standard errors and narrower 95% Wald Confidence Intervals (95% CI) in the analysis of all patients and stage III/IV cancer patients (Tables 1 and 2). The inclusion of the unexposed cluster also altered the intervention effect point estimates, although the 95% CI’s of the analyses with and without the unexposed cluster continued to overlap substantially. Furthermore, the differences between the point estimates with and without the unexposed cluster were small compared to the corresponding SE’s (Tables 1 and 2).

**Table 1:** Intervention effect estimates on hospital readmission

Analysis	Patients	$\:\widehat{\delta\:}$	SE	95% CI	OR 95% CI
$-$ unexposed cluster	All	0.053	0.176	(-0.294, 0.398)	(0.745, 1.489)
$+$ unexposed cluster	All	-0.017	0.154	(-0.319, 0.286)	(0.827, 1.331)
$-$ unexposed cluster	Stage III/IV	0.105	0.185	(-0.259, 0.468)	(0.772, 1.597)
$+$ unexposed cluster	Stage III/IV	0.016	0.163	(-0.304, 0.336)	(0.738, 1.400)

Intervention effect estimates $\widehat{\delta}$in analyses excluding ($-$) and including ($+$) the unexposed cluster in a fixed effects Logistic Regression analysis of data from a SW-CRT testing the effect of a novel co-rounding model of palliative care on hospital readmission within 30 days. The intervention effect $\widehat{\delta}$, standard errors, and 95% Wald Confidence Intervals for both $\widehat{\delta}$ and the Odds Ratio $(\text{O}\text{R}={e}^{\widehat{\delta}})$ were estimated for all cancer patients or only Stage III/IV cancer patients

**Table 2:** Intervention effect estimates on use of specialist palliative care

Analysis	Patients	$\:\widehat{\delta\:}$	SE	95% CI	OR 95% CI
$-$ unexposed cluster	All	0.019	0.202	(-0.379, 0.414)	(0.685, 1.512)
$+$ unexposed cluster	All	0.043	0.177	(-0.304, 0.391)	(0.738, 1.479)
$-$ unexposed cluster	Stage III/IV	-0.055	0.209	(-0.467, 0.354)	(0.627, 1.424)
$+$ unexposed cluster	Stage III/IV	-0.015	0.183	(-0.374, 0.345)	(0.688, 1.412)

Intervention effect estimates $\widehat{\delta}$ in analyses excluding ($-$) and including ($+$) the unexposed cluster in a fixed effects Logistic Regression analysis of data from a SW-CRT testing the effect of a novel co-rounding model of palliative care on use of specialist palliative care. The intervention effect $\widehat{\delta}$, standard errors, and 95% Wald Confidence Intervals for both $\widehat{\delta}$ and the Odds Ratio $(\text{O}\text{R}={e}^{\widehat{\delta}})$ were estimated for all cancer patients or only Stage III/IV cancer patients

## Discussion

Randomization removes covariate imbalance in expectation. However, it does not guarantee removal of covariate imbalance in a single randomized trial [10]. In a SW-CRT, mixed effects models are unable to control for unmeasured cluster-level confounders [4–8]. Furthermore, mixed effect analysis can have inflated type I error rates when the number of clusters is small [14, 15]. In contrast, the fixed effects model uses within-cluster comparisons to control for all unmeasured cluster-level time-invariant confounders [5] and do not exhibit this inflated type I error rate [14].

SW-CRTs may conclude with unexposed clusters (clusters never exposed to intervention) in real world research, such as when SW-CRTs are terminated early [23] or by design [24, 25]. Some previous publications on fixed effects and other methods that make within-unit comparisons have given the impression that unexposed units (in this case, clusters) should not be included in the analysis [5, 6, 22]. It has recently been proven that including an unexposed cluster will improve the precision of the intervention effect estimator in fixed effects analysis of SW-CRTs with continuous outcomes [26]. However, given that more than half of the published SW-CRT articles covered in a 2016 review had binary outcomes [12], it was important to extend our previous findings to SW-CRTs with non-continuous outcomes.

We mathematically proved that the inclusion of unexposed clusters in a fixed effects logistic and Poisson regression improves the precision of the intervention effect estimator. Furthermore, we prove that the gain in precision from including an unexposed cluster is equivalent to including an always-exposed cluster (Online Supplementary Material S3). We then demonstrated this improvement with simulated data, and data collected from a SW-CRT of a novel palliative care model. Our simulation results indicated that including unexposed clusters improves the precision, power, and reduces the RMSE of the fixed effects intervention effect estimator for both binary and count outcomes.

Our simulation results reveal that the precision gain of including some unexposed clusters was roughly equal to the precision gain of randomizing those clusters to receive the intervention in both the fixed effects logistic and Poisson regression. A similar result was observed with continuous outcomes [26]. Given that including an unexposed cluster is equivalent to including an always-exposed cluster, some parallels can be observed between this finding and the “hybrid” [24, 25] and “optimized” design [25]. The hybrid design was developed around the H&H mixed effects model and includes blocks of always-exposed and unexposed clusters [24, 25]. The SW-CRT designs described in this article can be interpreted as an example of the hybrid design. Future studies can mathematically compare the efficiency gain of including always-exposed and unexposed clusters against randomizing those clusters into a standard SW-CRT in a fixed effects model, and compare the properties of “hybrid” and “optimized” designs between fixed effects and mixed effects models.

Unexposed clusters should only be included if they arise from the same study population as exposed clusters. In the example of a seasonal malaria chemoprevention trial that was terminated early due to interim analysis results [23], unexposed clusters were clusters that were randomized to implement the interventions at later time periods. In the palliative care case study, the unexposed cluster arose due to restructuring of the oncology teams and there was no expansion of study population or change in case-mix [31]. Accordingly, these unexposed clusters are expected to be comparable to the exposed clusters and may be included in the analysis. In contrast, researchers should carefully consider the comparability of unexposed clusters if they arise following expansion of service coverage.

In scenarios where unexposed clusters are clusters that were randomized but did not receive the intervention, such as the previously described seasonal malaria chemoprevention trial [23], researchers should clarify the reason for those clusters remaining unexposed and the potential for selection bias. In scenarios where unexposed clusters were not part of the randomization process, such as the palliative care case study described in this article, researchers should exercise caution when considering whether to include these unexposed clusters in the analysis of such scenarios. Notably, the fixed effects model automatically adjusts for all measured and unmeasured cluster-level time-invariant confounding, therefore concern over biases due to the inclusion of a non-randomized unexposed cluster should be reduced. Similar concerns over the lack of randomization have previously motivated the use of the fixed effects model in the analysis of stepped-wedge designs [20, 33]. However, bias resulting from the inclusion of an unexposed cluster can still arise if the unexposed cluster has differing underlying period effects than the other clusters in the trial, hence the earlier emphasis on only including unexposed clusters that arise from the same study population.

Both the H&H mixed effects model and fixed effects model operate under the assumption that clusters have the same underlying period effects [11, 32]. This is especially important when including an unexposed cluster in a fixed effects model. In the palliative care analysis, we used AIC and BIC to determine whether including interaction terms between an unexposed cluster and period effects improved the model fit. The operating characteristics of these and other methods can be explored and validated in future work.

The fixed effects model and simulation data generating process described in this article both assume that observations are exchangeable between periods within clusters. However, this assumption does not always hold. Accordingly, mixed effects models with more complicated correlation structures have been proposed [3], including the “Hooper-Girling” mixed effects model, which includes an additional cluster-period random interaction term to induce a nested-exchangeable correlation structure [24, 34]. We note that the fixed effects model can be easily extended to have a similar nested correlation structure between periods within clusters by including a cluster-period random interaction term alongside the cluster fixed effect. The properties of such a nested fixed effects model have not yet been explicitly evaluated in the context of SW-CRTs. Still, we expect the results described here regarding the inclusion of unexposed clusters to similarly apply to such a nested fixed effects model with a cluster-period random interaction term; this can be validated in future work.

The CONSORT extension for SW-CRTs suggests reporting the intraclass correlation coefficient (ICC) estimates to help inform future studies [35]. Unlike mixed effects models, the fixed effects model does not automatically estimate the between-cluster variance [8]. However, Wooldridge [8] suggested that the sample variance of the cluster fixed effects estimates may be used to estimate the between-cluster variance. This has not been validated in the context of fixed effects analyses for SW-CRTs and should be explored in future work.

Using the dummy variable method for a logistic-regression fixed effects models, as we have here, has been discouraged in some texts due to the incidental parameter problem [5]. However, this is only an issue when the number of parameters increases at the same rate as the sample size [5]. This is not an issue in a SW-CRT, where clusters are the fixed effects units, with multiple individuals in each cluster-period cell.

## Conclusion

Overall, cross-sectional SW-CRTs with binary or count outcomes may conclude with unexposed or always-exposed clusters in scenarios where a fixed effects model may be preferred. In this article, we demonstrate that the inclusion of these clusters in the fixed effects analysis can lead to the improvements in precision, power, and RMSE of the fixed effects intervention effect estimator for SW-CRTs with binary or count outcomes.

## Supplementary Information

Additional File 1. Online Supplementary Materials S1 to S6

Additional File 2. R code for simulation, analysis, and plotting

## Data Availability

No datasets were generated or analysed during the current study.

## Abbreviations

CRT: Cluster randomized trial
SW-CRT: Stepped-wedge cluster randomized trial
H&H: Hussey and Hughes
ICC: Intra-cluster correlation coefficient
Abs Bias: Absolute bias
Rel Bias: Relative bias
CP: Coverage probability
SE: Standard error
RMSE: Root mean square error
AIC: Akaike Information Criterion
BIC: Bayesian Information Criterion
