# Supplementary material for: Inclusion of unexposed clusters improves the precision of fixed effects analysis of stepped-wedge cluster randomized trials with binary and count outcomes
Source: BMC Med Res Methodol. 2024 Oct 28;24:254. doi: 10.1186/s12874-024-02379-z (PMC11514785; doi:10.1186/s12874-024-02379-z)

Online Supplementary Material S1. Product matrices involved in the Proof of $\mathrm{Var}(\breve{\delta}) < \mathrm{Var}(\tilde{\delta})$

S1a. $\breve{X}'\breve{V}\breve{X}$, which includes the unexposed cluster (Cluster 5). Where $v_{ij} = \sum_{k=1}^{n_{ij}} v(\mu_{ijk})$ is the marginal sum of $v(\mu_{ijk})$ for each cluster:period cell.

$\breve{X}'\breve{V}\breve{X} =$

	Intervention	Period 2	Period 3	Period 4	Period 5	Cluster 1	Cluster 2	Cluster 3	Cluster 4	Cluster 5
I	$\sum_{j=2}^{5} v_{1,j} + \sum_{j=3}^{5} v_{2,j} + \sum_{j=4}^{5} v_{3,j} + v_{4,5}$	$v_{1,2}$	$\sum_{i=1}^{2} v_{i,3}$	$\sum_{i=1}^{3} v_{i,4}$	$\sum_{i=1}^{4} v_{i,5}$	$\sum_{j=2}^{5} v_{1,j}$	$\sum_{j=3}^{5} v_{2,j}$	$\sum_{j=4}^{5} v_{3,j}$	$v_{4,5}$	0
P2	$v_{1,2}$	$\sum_{i=1}^{5} v_{i,2}$	0	0	0	$v_{1,2}$	$v_{2,2}$	$v_{3,2}$	$v_{4,2}$	$v_{5,2}$
P3	$\sum_{i=1}^{2} v_{i,3}$	0	$\sum_{i=1}^{5} v_{i,3}$	0	0	$v_{1,3}$	$v_{2,3}$	$v_{3,3}$	$v_{4,3}$	$v_{5,3}$
P4	$\sum_{i=1}^{3} v_{i,4}$	0	0	$\sum_{i=1}^{5} v_{i,4}$	0	$v_{1,4}$	$v_{2,4}$	$v_{3,4}$	$v_{4,4}$	$v_{5,4}$
P5	$\sum_{i=1}^{4} v_{i,5}$	0	0	0	$\sum_{i=1}^{5} v_{i,5}$	$v_{1,5}$	$v_{2,5}$	$v_{3,5}$	$v_{4,5}$	$v_{5,5}$
C1	$\sum_{j=2}^{5} v_{1,j}$	$v_{1,2}$	$v_{1,3}$	$v_{1,4}$	$v_{1,5}$	$\sum_{j=1}^{5} v_{1,j}$	0	0	0	0
C2	$\sum_{j=3}^{5} v_{2,j}$	$v_{2,2}$	$v_{2,3}$	$v_{2,4}$	$v_{2,5}$	0	$\sum_{j=1}^{5} v_{2,j}$	0	0	0
C3	$\sum_{j=4}^{5} v_{3,j}$	$v_{3,2}$	$v_{3,3}$	$v_{3,4}$	$v_{3,5}$	0	0	$\sum_{j=1}^{5} v_{3,j}$	0	0
C4	$v_{4,5}$	$v_{4,2}$	$v_{4,3}$	$v_{4,4}$	$v_{4,5}$	0	0	0	$\sum_{j=1}^{5} v_{4,j}$	0
C5	0	$v_{5,2}$	$v_{5,3}$	$v_{5,4}$	$v_{5,5}$	0	0	0	0	$\sum_{j=1}^{5} v_{5,j}$

S1b. $\dot{X}'\dot{V}\dot{X}$, which excludes the unexposed cluster (Cluster 5). Where $v_{ij} = \sum_{k=1}^{n_{ij}} v(\mu_{ijk})$ is the marginal sum of $v(\mu_{ijk})$ for each cluster:period cell.

$\dot{X}'\dot{V}\dot{X} =$

	Intervention	Period 2	Period 3	Period 4	Period 5	Cluster 1	Cluster 2	Cluster 3	Cluster 4
I	$\sum_{j=2}^{5} v_{1,j} + \sum_{j=3}^{5} v_{2,j} + \sum_{j=4}^{5} v_{3,j} + v_{4,5}$	$v_{1,2}$	$\sum_{i=1}^{2} v_{i,3}$	$\sum_{i=1}^{3} v_{i,4}$	$\sum_{i=1}^{4} v_{i,5}$	$\sum_{j=2}^{5} v_{1,j}$	$\sum_{j=3}^{5} v_{2,j}$	$\sum_{j=4}^{5} v_{3,j}$	$v_{4,5}$
P2	$v_{1,2}$	$\sum_{i=1}^{4} v_{i,2}$	0	0	0	$v_{1,2}$	$v_{2,2}$	$v_{3,2}$	$v_{4,2}$
P3	$\sum_{i=1}^{2} v_{i,3}$	0	$\sum_{i=1}^{4} v_{i,3}$	0	0	$v_{1,3}$	$v_{2,3}$	$v_{3,3}$	$v_{4,3}$
P4	$\sum_{i=1}^{3} v_{i,4}$	0	0	$\sum_{i=1}^{4} v_{i,4}$	0	$v_{1,4}$	$v_{2,4}$	$v_{3,4}$	$v_{4,4}$
P5	$\sum_{i=1}^{4} v_{i,5}$	0	0	0	$\sum_{i=1}^{5} v_{i,5}$	$v_{1,5}$	$v_{2,5}$	$v_{3,5}$	$v_{4,5}$
C1	$\sum_{j=2}^{5} v_{1,j}$	$v_{1,2}$	$v_{1,3}$	$v_{1,4}$	$v_{1,5}$	$\sum_{j=1}^{5} v_{1,j}$	0	0	0
C2	$\sum_{j=3}^{5} v_{2,j}$	$v_{2,2}$	$v_{2,3}$	$v_{2,4}$	$v_{2,5}$	0	$\sum_{j=1}^{5} v_{2,j}$	0	0
C3	$\sum_{j=4}^{5} v_{3,j}$	$v_{3,2}$	$v_{3,3}$	$v_{3,4}$	$v_{3,5}$	0	0	$\sum_{j=1}^{5} v_{3,j}$	0
C4	$v_{4,5}$	$v_{4,2}$	$v_{4,3}$	$v_{4,4}$	$v_{4,5}$	0	0	0	$\sum_{j=1}^{5} v_{4,j}$

S1c. $\tilde{X}'\tilde{V}\tilde{X}$, which includes all periods in all exposed clusters and only the first period of the unexposed cluster (cluster 5). Where $v_{ij} = \sum_{k=1}^{n_{ij}} v(\mu_{ijk})$ is the marginal sum of $v(\mu_{ijk})$ for each cluster:period cell.

$\tilde{X}'\tilde{V}\tilde{X} =$

	Intervention	Period 2	Period 3	Period 4	Period 5	Cluster 1	Cluster 2	Cluster 3	Cluster 4	Cluster 5
I	$\sum_{j=2}^{5} v_{1,j} + \sum_{j=3}^{5} v_{2,j} + \sum_{j=4}^{5} v_{3,j} + v_{4,5}$	$v_{1,2}$	$\sum_{i=1}^{2} v_{i,3}$	$\sum_{i=1}^{3} v_{i,4}$	$\sum_{i=1}^{4} v_{i,5}$	$\sum_{j=2}^{5} v_{1,j}$	$\sum_{j=3}^{5} v_{2,j}$	$\sum_{j=4}^{5} v_{3,j}$	$v_{4,5}$	0
P2	$v_{1,2}$	$\sum_{i=1}^{4} v_{i,2}$	0	0	0	$v_{1,2}$	$v_{2,2}$	$v_{3,2}$	$v_{4,2}$	0
P3	$\sum_{i=1}^{2} v_{i,3}$	0	$\sum_{i=1}^{4} v_{i,3}$	0	0	$v_{1,3}$	$v_{2,3}$	$v_{3,3}$	$v_{4,3}$	0
P4	$\sum_{i=1}^{3} v_{i,4}$	0	0	$\sum_{i=1}^{4} v_{i,4}$	0	$v_{1,4}$	$v_{2,4}$	$v_{3,4}$	$v_{4,4}$	0
P5	$\sum_{i=1}^{4} v_{i,5}$	0	0	0	$\sum_{i=1}^{5} v_{i,5}$	$v_{1,5}$	$v_{2,5}$	$v_{3,5}$	$v_{4,5}$	0
C1	$\sum_{j=2}^{5} v_{1,j}$	$v_{1,2}$	$v_{1,3}$	$v_{1,4}$	$v_{1,5}$	$\sum_{j=1}^{5} v_{1,j}$	0	0	0	0
C2	$\sum_{j=3}^{5} v_{2,j}$	$v_{2,2}$	$v_{2,3}$	$v_{2,4}$	$v_{2,5}$	0	$\sum_{j=1}^{5} v_{2,j}$	0	0	0
C3	$\sum_{j=4}^{5} v_{3,j}$	$v_{3,2}$	$v_{3,3}$	$v_{3,4}$	$v_{3,5}$	0	0	$\sum_{j=1}^{5} v_{3,j}$	0	0
C4	$v_{4,5}$	$v_{4,2}$	$v_{4,3}$	$v_{4,4}$	$v_{4,5}$	0	0	0	$\sum_{j=1}^{5} v_{4,j}$	0
C5	0	0	0	0	0	0	0	0	0	$v_{5,1}$

Online Supplementary Material S2. Complete Proof of $\mathrm{Var}\big(\breve{\boldsymbol{\delta}}\big) < \mathrm{Var}\big(\tilde{\boldsymbol{\delta}}\big)$.

First, we represent $\breve{X}'\breve{V}\breve{X}$ (S1a) and $\tilde{X}'\tilde{V}\tilde{X}$ (S1c) in terms of submatrices:

$$\breve{X}'\breve{V}\breve{X} = \begin{pmatrix} \breve{A} & \breve{B}' \\ \breve{B} & \breve{D} \end{pmatrix},$$

$$\tilde{X}'\tilde{V}\tilde{X} = \begin{pmatrix} \tilde{A} & \tilde{B}' \\ \tilde{B} & \tilde{D} \end{pmatrix}.$$

Submatrix $\breve{A} = \tilde{A}$ is a scalar equal to the sum of the weights $v\big(\mu_{ijk}\big)$ for all exposed participants. Submatrix $\breve{B} = \tilde{B}$ is the 9 by 1 vector of marginal weighted sums for participants who receive the intervention in each period and cluster. Submatrices $\breve{D}$ and $\tilde{D}$ represent the 9 by 9 weighted matrix product of the rows and columns of the design matrix that represent the dummy variables for the periods and clusters. Accordingly:

$$\mathrm{Var}\big(\breve{\delta}\big) = (\breve{X}'\breve{V}\breve{X})^{-1}_{1,1} = (\breve{A} - \breve{B}'\breve{D}^{-1}\breve{B})^{-1},$$

$$\mathrm{Var}\big(\tilde{\delta}\big) = (\tilde{X}'\tilde{V}\tilde{X})^{-1}_{1,1} = (\tilde{A} - \tilde{B}'\tilde{D}^{-1}\tilde{B})^{-1}.$$

Given that $\breve{X}'\breve{V}\breve{X}$ and $\tilde{X}'\tilde{V}\tilde{X}$ are same-sized positive definite matrices ($\breve{X}'\breve{V}\breve{X} \succ 0$ and $\tilde{X}'\tilde{V}\tilde{X} \succ 0$), then their principal submatrices $\breve{D}$ and $\tilde{D}$ are also positive definite matrices ($\breve{D} \succ 0$ and $\tilde{D} \succ 0$) (Horn and Johnson 2017). The difference between $\breve{D}$ and $\tilde{D}$ is positive semi-definite, where $x'\big(\breve{D} - \tilde{D}\big)x \geq 0$ for all x in $\mathbb{R}^9$ (Horn and Johnson 2017). Therefore, $\breve{D}$ and $\tilde{D}$ can be ordered as induced by Loewner partial ordering (Horn and Johnson 2017):

$$\breve{D} \succcurlyeq \tilde{D},$$

and:

$$\breve{D}^{-1} \preccurlyeq \tilde{D}^{-1}.$$

Given that vector $\breve{B} = \tilde{B}$ (here on referred to as $\mathbb{B}$) and scalar $\breve{A} = \tilde{A}$ (here on referred to as $\mathbb{A}$), then:

$$\big(\mathbb{A} - \mathbb{B}'\breve{D}^{-1}\mathbb{B}\big)^{-1} \leq \big(\mathbb{A} - \mathbb{B}'\tilde{D}^{-1}\mathbb{B}\big)^{-1},$$

and:

$$\mathrm{Var}\big(\breve{\delta}\big) \leq \mathrm{Var}\big(\tilde{\delta}\big).$$

Assume that $\mathrm{Var}\big(\breve{\delta}\big) = \mathrm{Var}\big(\tilde{\delta}\big)$. This implies that:

$$\mathbb{B}'\breve{D}^{-1}\mathbb{B} = \mathbb{B}'\tilde{D}^{-1}\mathbb{B}$$
$$\mathbb{B}'\big(\tilde{D}^{-1} - \breve{D}^{-1}\big)\mathbb{B}$$

Since $x'Ax = 0$ iff $Ax = 0$ [Observation 7.1.6] (Horn and Johnson 2017), the above statement reduces to:

$$\big(\tilde{D}^{-1} - \breve{D}^{-1}\big)\mathbb{B} = 0$$

$\breve{D}$ and $\widetilde{D}$ are further broken down into submatrices, where $D3$ corresponds with observations from the unexposed cluster:

$\breve{D} =$

$$
\begin{array}{c|ccccccccc|}
 & \text{P2} & \text{P3} & \text{P4} & \text{P5} & \text{C1} & \text{C2} & \text{C3} & \text{C4} & \text{C5} \\
\text{P2} & \sum_{i=1}^{5} v_{i,2} & 0 & 0 & 0 & v_{1,2} & v_{2,2} & v_{3,2} & v_{4,2} & v_{5,2} \\
\text{P3} & 0 & \sum_{i=1}^{5} v_{i,3} & 0 & 0 & v_{1,3} & v_{2,3} & v_{3,3} & v_{4,3} & v_{5,3} \\
\text{P4} & 0 & 0 & \sum_{i=1}^{5} v_{i,4} & 0 & v_{1,4} & v_{2,4} & v_{3,4} & v_{4,4} & v_{5,4} \\
\text{P5} & 0 & 0 & 0 & \sum_{i=1}^{5} v_{i,5} & v_{1,5} & v_{2,5} & v_{3,5} & v_{4,5} & v_{5,5} \\
\text{C1} & v_{1,2} & v_{1,3} & v_{1,4} & v_{1,5} & \sum_{j=1}^{5} v_{1,j} & 0 & 0 & 0 & 0 \\
\text{C2} & v_{2,2} & v_{2,3} & v_{2,4} & v_{2,5} & 0 & \sum_{j=1}^{5} v_{2,j} & 0 & 0 & 0 \\
\text{C3} & v_{3,2} & v_{3,3} & v_{3,4} & v_{3,5} & 0 & 0 & \sum_{j=1}^{5} v_{3,j} & 0 & 0 \\
\text{C4} & v_{4,2} & v_{4,3} & v_{4,4} & v_{4,5} & 0 & 0 & 0 & \sum_{j=1}^{5} v_{4,j} & 0 \\
\text{C5} & v_{5,2} & v_{5,3} & v_{5,4} & v_{5,5} & 0 & 0 & 0 & 0 & \sum_{j=1}^{5} v_{5,j}
\end{array}
$$

$$= \begin{pmatrix} \breve{D1} & \breve{D2} \\ \breve{D2}' & \breve{D3} \end{pmatrix}$$

$\widetilde{D} =$

$$
\begin{array}{c|ccccccccc|}
 & \text{P2} & \text{P3} & \text{P4} & \text{P5} & \text{C1} & \text{C2} & \text{C3} & \text{C4} & \text{C5} \\
\text{P2} & \sum_{i=1}^{4} v_{i,2} & 0 & 0 & 0 & v_{1,2} & v_{2,2} & v_{3,2} & v_{4,2} & 0 \\
\text{P3} & 0 & \sum_{i=1}^{4} v_{i,3} & 0 & 0 & v_{1,3} & v_{2,3} & v_{3,3} & v_{4,3} & 0 \\
\text{P4} & 0 & 0 & \sum_{i=1}^{4} v_{i,4} & 0 & v_{1,4} & v_{2,4} & v_{3,4} & v_{4,4} & 0 \\
\text{P5} & 0 & 0 & 0 & \sum_{i=1}^{5} v_{i,5} & v_{1,5} & v_{2,5} & v_{3,5} & v_{4,5} & 0 \\
\text{C1} & v_{1,2} & v_{1,3} & v_{1,4} & v_{1,5} & \sum_{j=1}^{5} v_{1,j} & 0 & 0 & 0 & 0 \\
\text{C2} & v_{2,2} & v_{2,3} & v_{2,4} & v_{2,5} & 0 & \sum_{j=1}^{5} v_{2,j} & 0 & 0 & 0 \\
\text{C3} & v_{3,2} & v_{3,3} & v_{3,4} & v_{3,5} & 0 & 0 & \sum_{j=1}^{5} v_{3,j} & 0 & 0 \\
\text{C4} & v_{4,2} & v_{4,3} & v_{4,4} & v_{4,5} & 0 & 0 & 0 & \sum_{j=1}^{5} v_{4,j} & 0 \\
\text{C5} & 0 & 0 & 0 & 0 & 0 & 0 & 0 & 0 & v_{5,1}
\end{array}
$$

$$= \begin{pmatrix} \widetilde{D1} & \widetilde{D2} \\ \widetilde{D2}' & \widetilde{D3} \end{pmatrix} = \begin{pmatrix} \widetilde{D1} & 0 \\ 0 & \widetilde{D3} \end{pmatrix}$$

With inverses:

$$\breve{D}^{-1} = \begin{pmatrix} \left(\breve{D1} - \breve{D2}\breve{D3}^{-1}\breve{D2}'\right)^{-1} & -\left(\breve{D1} - \breve{D2}\breve{D3}^{-1}\breve{D2}'\right)^{-1}\breve{D2}\breve{D3}^{-1} \\ -\breve{D3}^{-1}\breve{D2}'\left(\breve{D1} - \breve{D2}\breve{D3}^{-1}\breve{D2}'\right)^{-1} & \breve{D3}^{-1} + \breve{D3}^{-1}\breve{D2}'\left(\breve{D1} - \breve{D2}\breve{D3}^{-1}\breve{D2}'\right)^{-1}\breve{D2}\breve{D3}^{-1} \end{pmatrix}$$

$$\widetilde{D}^{-1} = \begin{pmatrix} \widetilde{D1}^{-1} & 0 \\ 0 & \widetilde{D3}^{-1} \end{pmatrix}.$$

Recall that $\mathbb{B} = \breve{B} = \tilde{B} = (\dot{B}, 0)$, then $(\widetilde{D}^{-1} - \breve{D}^{-1})\mathbb{B} = 0$ can then be rewritten as:

$$\begin{pmatrix} \widetilde{D1}^{-1} - \left(\breve{D1} - \breve{D2}\breve{D3}^{-1}\breve{D2}'\right)^{-1} & 0 + \left(\breve{D1} - \breve{D2}\breve{D3}^{-1}\breve{D2}'\right)^{-1}\breve{D2}\breve{D3}^{-1} \\ 0 + \breve{D3}^{-1}\breve{D2}'\left(\breve{D1} - \breve{D2}\breve{D3}^{-1}\breve{D2}'\right)^{-1} & \widetilde{D3}^{-1} - \left(\breve{D3}^{-1} + \breve{D3}^{-1}\breve{D2}'\left(\breve{D1} - \breve{D2}\breve{D3}^{-1}\breve{D2}'\right)^{-1}\breve{D2}\breve{D3}^{-1}\right) \end{pmatrix} \begin{pmatrix} \dot{B} \\ 0 \end{pmatrix} = 0.$$

Leading to the following statement which cannot be true:

$$\widetilde{D3}^{-1}\widetilde{D2}'\left(\widetilde{D1} - \widetilde{D2}\widetilde{D3}^{-1}\widetilde{D2}'\right)^{-1}\dot{B} = 0.$$

Since $\check{X}'\check{V}\check{X}$ is positive definite, then $\check{D}$ is positive definite [Observation 7.1.2] (Horn and Johnson 2017). Therefore $\check{D}^{-1}$ is also positive definite [Observation 7.2.1] (Horn and Johnson 2017). Given that $\check{D}^{-1}$ is positive definite, $\left(\widetilde{D1} - \widetilde{D2}\widetilde{D3}^{-1}\widetilde{D2}'\right)^{-1}$ is also positive definite (as a principal submatrix of $\check{D}^{-1}$) [Observation 7.1.2] (Horn and Johnson 2017). By definition, $x'\left(\widetilde{D1} - \widetilde{D2}\widetilde{D3}^{-1}\widetilde{D2}'\right)^{-1}x > 0$ unless x is a zero vector.

Furthermore, $\widetilde{D3}^{-1}\widetilde{D2}' = (\frac{v_{5,2}}{\sum_{j=1}^{5} v_{5,j}},\ \frac{v_{5,3}}{\sum_{j=1}^{5} v_{5,j}},\ \frac{v_{5,4}}{\sum_{j=1}^{5} v_{5,j}},\ \frac{v_{5,5}}{\sum_{j=1}^{5} v_{5,j}},\ 0,\ 0,\ 0,\ 0)$ which contains elements > 0. Altogether:

$$\widetilde{D3}^{-1}\widetilde{D2}'\left(\widetilde{D1} - \widetilde{D2}\widetilde{D3}^{-1}\widetilde{D2}'\right)^{-1} \neq 0.$$

Additionally, all elements of $\dot{B} > 0$. Therefore, we have a contradiction where:

$$\widetilde{D3}^{-1}\widetilde{D2}'\left(\widetilde{D1} - \widetilde{D2}\widetilde{D3}^{-1}\widetilde{D2}'\right)^{-1}\dot{B} \neq 0.$$

This satisfies our proof by contradiction, revealing that the initial assumption $\mathrm{Var}(\check{\delta}) = \mathrm{Var}(\tilde{\delta})$ is false and $\mathrm{Var}(\check{\delta}) \neq \mathrm{Var}(\tilde{\delta})$. This combined with our previously derived inequality $\mathrm{Var}(\check{\delta}) \leq \mathrm{Var}(\tilde{\delta})$ proves $\mathrm{Var}(\check{\delta}) < \mathrm{Var}(\tilde{\delta})$.

References

Horn, Roger A., and Charles R. Johnson. 2017. *Matrix Analysis*. Second edition, Corrected reprint. New York, NY: Cambridge University Press.

Online Supplementary Material S3. The effect of including an always-exposed cluster on the precision of the intervention effect estimator in a SW-CRT with binary or count outcomes.

The variance of the intervention effect estimator is equivalent when including a cluster that is either unexposed or always-exposed. Let $\bar{\bar{X}}$ be the $\bar{\bar{N}} \times 10$ design matrix for the analysis containing observations from all 5 periods in all 5 clusters, where cluster 5 is an always-exposed cluster. Accordingly, $\bar{\bar{X}}'\bar{\bar{V}}\bar{\bar{X}}$ resembles the matrix product $\breve{X}'\breve{V}\breve{X}$ (S1a.) with an additional increase in the overall and marginal total of individuals that receive the intervention from the always-exposed cluster 5.

Where $v_{ij} = \sum_{k=1}^{n_{ij}} v(\mu_{ijk})$ is the marginal sum of $v(\mu_{ijk})$ for each cluster:period cell.

$$\bar{\bar{X}}'\bar{\bar{V}}\bar{\bar{X}} =$$

	Intervention	Period 2	Period 3	Period 4	Period 5	Cluster 1	Cluster 2	Cluster 3	Cluster 4	Cluster 5
I	$\sum_{j=2}^{5} v_{1,j} + \sum_{j=3}^{5} v_{2,j} + \sum_{j=4}^{5} v_{3,j} + v_{4,5} + \sum_{j=1}^{5} v_{5,j}$	$v_{1,2} + v_{5,2}$	$\sum_{i=1}^{2} v_{i,3} + v_{5,3}$	$\sum_{i=1}^{3} v_{i,4} + v_{5,4}$	$\sum_{i=1}^{4} v_{i,5} + v_{5,5}$	$\sum_{j=2}^{5} v_{1,j}$	$\sum_{j=3}^{5} v_{2,j}$	$\sum_{j=4}^{5} v_{3,j}$	$v_{4,5}$	$\sum_{j=1}^{5} v_{5,j}$
P2	$v_{1,2} + v_{5,2}$	$\sum_{i=1}^{5} v_{i,2}$	0	0	0	$v_{1,2}$	$v_{2,2}$	$v_{3,2}$	$v_{4,2}$	$v_{5,2}$
P3	$\sum_{i=1}^{2} v_{i,3} + v_{5,3}$	0	$\sum_{i=1}^{5} v_{i,3}$	0	0	$v_{1,3}$	$v_{2,3}$	$v_{3,3}$	$v_{4,3}$	$v_{5,3}$
P4	$\sum_{i=1}^{3} v_{i,4} + v_{5,4}$	0	0	$\sum_{i=1}^{5} v_{i,4}$	0	$v_{1,4}$	$v_{2,4}$	$v_{3,4}$	$v_{4,4}$	$v_{5,4}$
P5	$\sum_{i=1}^{4} v_{i,5} + v_{5,5}$	0	0	0	$\sum_{i=1}^{5} v_{i,5}$	$v_{1,5}$	$v_{2,5}$	$v_{3,5}$	$v_{4,5}$	$v_{5,5}$
C1	$\sum_{j=2}^{5} v_{1,j}$	$v_{1,2}$	$v_{1,3}$	$v_{1,4}$	$v_{1,5}$	$\sum_{j=1}^{5} v_{1,j}$	0	0	0	0
C2	$\sum_{j=3}^{5} v_{2,j}$	$v_{2,2}$	$v_{2,3}$	$v_{2,4}$	$v_{2,5}$	0	$\sum_{j=1}^{5} v_{2,j}$	0	0	0
C3	$\sum_{j=4}^{5} v_{3,j}$	$v_{3,2}$	$v_{3,3}$	$v_{3,4}$	$v_{3,5}$	0	0	$\sum_{j=1}^{5} v_{3,j}$	0	0
C4	$v_{4,5}$	$v_{4,2}$	$v_{4,3}$	$v_{4,4}$	$v_{4,5}$	0	0	0	$\sum_{j=1}^{5} v_{4,j}$	0
C5	$\sum_{j=1}^{5} v_{5,j}$	$v_{5,2}$	$v_{5,3}$	$v_{5,4}$	$v_{5,5}$	0	0	0	0	$\sum_{j=1}^{5} v_{5,j}$

The specified submatrices below are changed from those specified in the main text,

$$\breve{X}'\breve{V}\breve{X} = \begin{pmatrix} \breve{\Omega}_{11} & \breve{\Omega}_{12} \\ \breve{\Omega}_{21} & \breve{\Omega}_{22} \end{pmatrix}$$

$$\bar{\bar{X}}'\bar{\bar{V}}\bar{\bar{X}} = \begin{pmatrix}\bar{\bar{\Omega}}_{11} & \bar{\bar{\Omega}}_{12}\\ \bar{\bar{\Omega}}_{21} & \bar{\bar{\Omega}}_{22}\end{pmatrix}$$

where submatrix $\breve{\Omega}_{11}$ is now a weighted 9×9 matrix product of the rows of $\check{X}'$ and columns of $\check{X}$ that represent the dummy variables for all model variables besides cluster 5. Submatrix $\breve{\Omega}_{21} = \breve{\Omega}_{12}'$ is a row vector of length 9 that equals the weighted dot product of the last row of $\check{X}'$ (indicating cluster 5) by all columns but the last of $\check{X}$ (containing dummy variables for all model variables besides cluster 5). Submatrix $\breve{\Omega}_{22}$ is a scalar that equals the weighted dot product of the last row of $\check{X}'$ by the last column of $\check{X}$, representing the total number of trial participants in cluster 5, where $\breve{\Omega}_{22} = \bar{\bar{\Omega}}_{22} = \sum_{j=1}^{5} v_{5,j}$.

Under these specified submatrices, $\mathrm{Var}(\check{\delta}) = (\breve{\Omega}_{11} - \breve{\Omega}_{12}\breve{\Omega}_{22}^{-1}\breve{\Omega}_{21})_{1,1}^{-1}$ and $\mathrm{Var}(\bar{\bar{\delta}}) = (\bar{\bar{\Omega}}_{11} - \bar{\bar{\Omega}}_{12}\bar{\bar{\Omega}}_{22}^{-1}\bar{\bar{\Omega}}_{21})_{1,1}^{-1}$ are shown below to be equal.

$$\mathrm{Var}(\check{\delta}) = (\breve{\Omega}_{11} - \breve{\Omega}_{12}\breve{\Omega}_{22}^{-1}\breve{\Omega}_{21})_{1,1}^{-1}$$

$$= \left(\breve{\Omega}_{11} - \breve{\Omega}_{12}\frac{1}{\sum_{j=1}^{5} v_{5,j}}\breve{\Omega}_{21}\right)_{1,1}^{-1}$$

$$= \left(\breve{\Omega}_{11} - \begin{pmatrix}
0 & 0 & 0 & 0 & 0 & 0 & 0 & 0 & 0\\
0 & \frac{v_{5,2}^2}{\sum_{j=1}^{5} v_{5,j}} & \frac{v_{5,2}v_{5,3}}{\sum_{j=1}^{5} v_{5,j}} & \frac{v_{5,2}v_{5,4}}{\sum_{j=1}^{5} v_{5,j}} & \frac{v_{5,2}v_{5,5}}{\sum_{j=1}^{5} v_{5,j}} & 0 & 0 & 0 & 0\\
0 & \frac{v_{5,3}v_{5,2}}{\sum_{j=1}^{5} v_{5,j}} & \frac{v_{5,3}^2}{\sum_{j=1}^{5} v_{5,j}} & \frac{v_{5,3}v_{5,4}}{\sum_{j=1}^{5} v_{5,j}} & \frac{v_{5,3}v_{5,5}}{\sum_{j=1}^{5} v_{5,j}} & 0 & 0 & 0 & 0\\
0 & \frac{v_{5,4}v_{5,2}}{\sum_{j=1}^{5} v_{5,j}} & \frac{v_{5,4}v_{5,3}}{\sum_{j=1}^{5} v_{5,j}} & \frac{v_{5,4}^2}{\sum_{j=1}^{5} v_{5,j}} & \frac{v_{5,4}v_{5,5}}{\sum_{j=1}^{5} v_{5,j}} & 0 & 0 & 0 & 0\\
0 & \frac{v_{5,5}v_{5,2}}{\sum_{j=1}^{5} v_{5,j}} & \frac{v_{5,5}v_{5,3}}{\sum_{j=1}^{5} v_{5,j}} & \frac{v_{5,5}v_{5,4}}{\sum_{j=1}^{5} v_{5,j}} & \frac{v_{5,5}^2}{\sum_{j=1}^{5} v_{5,j}} & 0 & 0 & 0 & 0\\
0 & 0 & 0 & 0 & 0 & 0 & 0 & 0 & 0\\
0 & 0 & 0 & 0 & 0 & 0 & 0 & 0 & 0\\
0 & 0 & 0 & 0 & 0 & 0 & 0 & 0 & 0\\
0 & 0 & 0 & 0 & 0 & 0 & 0 & 0 & 0
\end{pmatrix}\right)_{1,1}^{-1}$$

and,

$$\mathrm{Var}(\bar{\bar{\delta}}) = (\bar{\bar{\Omega}}_{11} - \bar{\bar{\Omega}}_{12}\bar{\bar{\Omega}}_{22}^{-1}\bar{\bar{\Omega}}_{21})_{1,1}^{-1}$$

$$= \left(\bar{\bar{\Omega}}_{11} - \bar{\bar{\Omega}}_{12}\frac{1}{\sum_{j=1}^{5} v_{5,j}}\bar{\bar{\Omega}}_{21}\right)_{1,1}^{-1}$$

$$= \left(\bar{\bar{\Omega}}_{11} - \begin{pmatrix} \sum_{j=1}^{5} v_{5,j} & v_{5,2} & v_{5,3} & v_{5,4} & v_{5,5} & 0 & 0 & 0 & 0 \\ v_{5,2} & \frac{v_{5,2}^2}{\sum_{j=1}^5 v_{5,j}} & \frac{v_{5,2}v_{5,3}}{\sum_{j=1}^5 v_{5,j}} & \frac{v_{5,2}v_{5,4}}{\sum_{j=1}^5 v_{5,j}} & \frac{v_{5,2}v_{5,5}}{\sum_{j=1}^5 v_{5,j}} & 0 & 0 & 0 & 0 \\ v_{5,3} & \frac{v_{5,3}v_{5,2}}{\sum_{j=1}^5 v_{5,j}} & \frac{v_{5,3}^2}{\sum_{j=1}^5 v_{5,j}} & \frac{v_{5,3}v_{5,4}}{\sum_{j=1}^5 v_{5,j}} & \frac{v_{5,3}v_{5,5}}{\sum_{j=1}^5 v_{5,j}} & 0 & 0 & 0 & 0 \\ v_{5,4} & \frac{v_{5,4}v_{5,2}}{\sum_{j=1}^5 v_{5,j}} & \frac{v_{5,4}v_{5,3}}{\sum_{j=1}^5 v_{5,j}} & \frac{v_{5,4}^2}{\sum_{j=1}^5 v_{5,j}} & \frac{v_{5,4}v_{5,5}}{\sum_{j=1}^5 v_{5,j}} & 0 & 0 & 0 & 0 \\ v_{5,5} & \frac{v_{5,5}v_{5,2}}{\sum_{j=1}^5 v_{5,j}} & \frac{v_{5,5}v_{5,3}}{\sum_{j=1}^5 v_{5,j}} & \frac{v_{5,5}v_{5,4}}{\sum_{j=1}^5 v_{5,j}} & \frac{v_{5,5}^2}{\sum_{j=1}^5 v_{5,j}} & 0 & 0 & 0 & 0 \\ 0 & 0 & 0 & 0 & 0 & 0 & 0 & 0 & 0 \\ 0 & 0 & 0 & 0 & 0 & 0 & 0 & 0 & 0 \\ 0 & 0 & 0 & 0 & 0 & 0 & 0 & 0 & 0 \\ 0 & 0 & 0 & 0 & 0 & 0 & 0 & 0 & 0 \end{pmatrix} \right)^{-1}_{1,1}$$

$$= \left(\bar{\bar{\Omega}}_{11} - \begin{pmatrix} \sum_{j=1}^{5} v_{5,j} & v_{5,2} & v_{5,3} & v_{5,4} & v_{5,5} & 0 & 0 & 0 & 0 \\ v_{5,2} & 0 & 0 & 0 & 0 & 0 & 0 & 0 & 0 \\ v_{5,3} & 0 & 0 & 0 & 0 & 0 & 0 & 0 & 0 \\ v_{5,4} & 0 & 0 & 0 & 0 & 0 & 0 & 0 & 0 \\ v_{5,5} & 0 & 0 & 0 & 0 & 0 & 0 & 0 & 0 \\ 0 & 0 & 0 & 0 & 0 & 0 & 0 & 0 & 0 \\ 0 & 0 & 0 & 0 & 0 & 0 & 0 & 0 & 0 \\ 0 & 0 & 0 & 0 & 0 & 0 & 0 & 0 & 0 \\ 0 & 0 & 0 & 0 & 0 & 0 & 0 & 0 & 0 \end{pmatrix} \right.$$

$$\left. - \begin{pmatrix} 0 & 0 & 0 & 0 & 0 & 0 & 0 & 0 & 0 \\ 0 & \frac{v_{5,2}^2}{\sum_{j=1}^5 v_{5,j}} & \frac{v_{5,2}v_{5,3}}{\sum_{j=1}^5 v_{5,j}} & \frac{v_{5,2}v_{5,4}}{\sum_{j=1}^5 v_{5,j}} & \frac{v_{5,2}v_{5,5}}{\sum_{j=1}^5 v_{5,j}} & 0 & 0 & 0 & 0 \\ 0 & \frac{v_{5,3}v_{5,2}}{\sum_{j=1}^5 v_{5,j}} & \frac{v_{5,3}^2}{\sum_{j=1}^5 v_{5,j}} & \frac{v_{5,3}v_{5,4}}{\sum_{j=1}^5 v_{5,j}} & \frac{v_{5,3}v_{5,5}}{\sum_{j=1}^5 v_{5,j}} & 0 & 0 & 0 & 0 \\ 0 & \frac{v_{5,4}v_{5,2}}{\sum_{j=1}^5 v_{5,j}} & \frac{v_{5,4}v_{5,3}}{\sum_{j=1}^5 v_{5,j}} & \frac{v_{5,4}^2}{\sum_{j=1}^5 v_{5,j}} & \frac{v_{5,4}v_{5,5}}{\sum_{j=1}^5 v_{5,j}} & 0 & 0 & 0 & 0 \\ 0 & \frac{v_{5,5}v_{5,2}}{\sum_{j=1}^5 v_{5,j}} & \frac{v_{5,5}v_{5,3}}{\sum_{j=1}^5 v_{5,j}} & \frac{v_{5,5}v_{5,4}}{\sum_{j=1}^5 v_{5,j}} & \frac{v_{5,5}^2}{\sum_{j=1}^5 v_{5,j}} & 0 & 0 & 0 & 0 \\ 0 & 0 & 0 & 0 & 0 & 0 & 0 & 0 & 0 \\ 0 & 0 & 0 & 0 & 0 & 0 & 0 & 0 & 0 \\ 0 & 0 & 0 & 0 & 0 & 0 & 0 & 0 & 0 \\ 0 & 0 & 0 & 0 & 0 & 0 & 0 & 0 & 0 \end{pmatrix} \right)^{-1}_{1,1}$$

$$
= \left(\breve{\Omega}_{11} - \begin{pmatrix} 0 & 0 & 0 & 0 & 0 & 0 & 0 & 0 & 0 \\ 0 & \frac{v_{5,2}^2}{\sum_{j=1}^{5} v_{5,j}} & \frac{v_{5,2} v_{5,3}}{\sum_{j=1}^{5} v_{5,j}} & \frac{v_{5,2} v_{5,4}}{\sum_{j=1}^{5} v_{5,j}} & \frac{v_{5,2} v_{5,5}}{\sum_{j=1}^{5} v_{5,j}} & 0 & 0 & 0 & 0 \\ 0 & \frac{v_{5,3} v_{5,2}}{\sum_{j=1}^{5} v_{5,j}} & \frac{v_{5,3}^2}{\sum_{j=1}^{5} v_{5,j}} & \frac{v_{5,3} v_{5,4}}{\sum_{j=1}^{5} v_{5,j}} & \frac{v_{5,3} v_{5,5}}{\sum_{j=1}^{5} v_{5,j}} & 0 & 0 & 0 & 0 \\ 0 & \frac{v_{5,4} v_{5,2}}{\sum_{j=1}^{5} v_{5,j}} & \frac{v_{5,4} v_{5,3}}{\sum_{j=1}^{5} v_{5,j}} & \frac{v_{5,4}^2}{\sum_{j=1}^{5} v_{5,j}} & \frac{v_{5,4} v_{5,5}}{\sum_{j=1}^{5} v_{5,j}} & 0 & 0 & 0 & 0 \\ 0 & \frac{v_{5,5} v_{5,2}}{\sum_{j=1}^{5} v_{5,j}} & \frac{v_{5,5} v_{5,3}}{\sum_{j=1}^{5} v_{5,j}} & \frac{v_{5,5} v_{5,4}}{\sum_{j=1}^{5} v_{5,j}} & \frac{v_{5,5}^2}{\sum_{j=1}^{5} v_{5,j}} & 0 & 0 & 0 & 0 \\ 0 & 0 & 0 & 0 & 0 & 0 & 0 & 0 & 0 \\ 0 & 0 & 0 & 0 & 0 & 0 & 0 & 0 & 0 \\ 0 & 0 & 0 & 0 & 0 & 0 & 0 & 0 & 0 \\ 0 & 0 & 0 & 0 & 0 & 0 & 0 & 0 & 0 \end{pmatrix} \right)^{-1}_{1,1}
$$

which is equal to the $\mathrm{Var}(\breve{\delta})$ specified above.

In conclusion, we demonstrate that whether cluster 5 is unexposed or always-exposed has no effect on the variance of the intervention effect estimator, and $\mathrm{Var}(\breve{\delta}) = \mathrm{Var}(\bar{\bar{\delta}})$.

Online Supplementary Material S4. Complete Simulation Results for Binary Outcomes. The impact of including unexposed clusters on properties of the fixed effects treatment effect estimator for binary outcomes, presented across number of exposed clusters, unexposed clusters, average cluster size $\mathrm{E}[n_i]$, different true treatment effect values δ, and between-cluster variance τ_{α}^{2}.

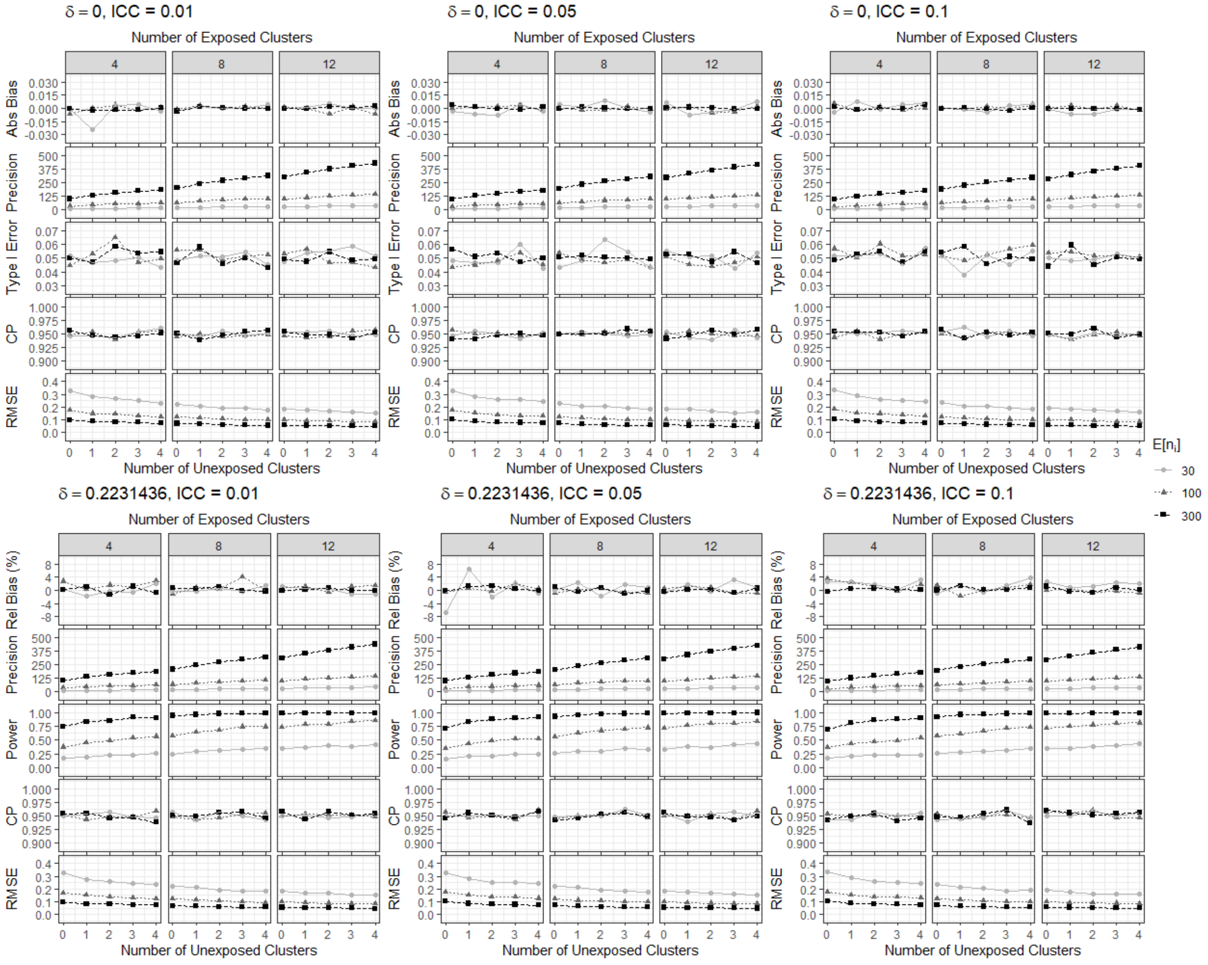

Online Supplementary Material S5. Complete Simulation Results for Count Outcomes. The impact of including unexposed clusters on properties of the fixed effects treatment effect estimator for count outcomes, presented across number of exposed clusters, unexposed clusters, average cluster size $\mathrm{E}[n_i]$, different true treatment effect values δ, and between-cluster variance τ_α^2.

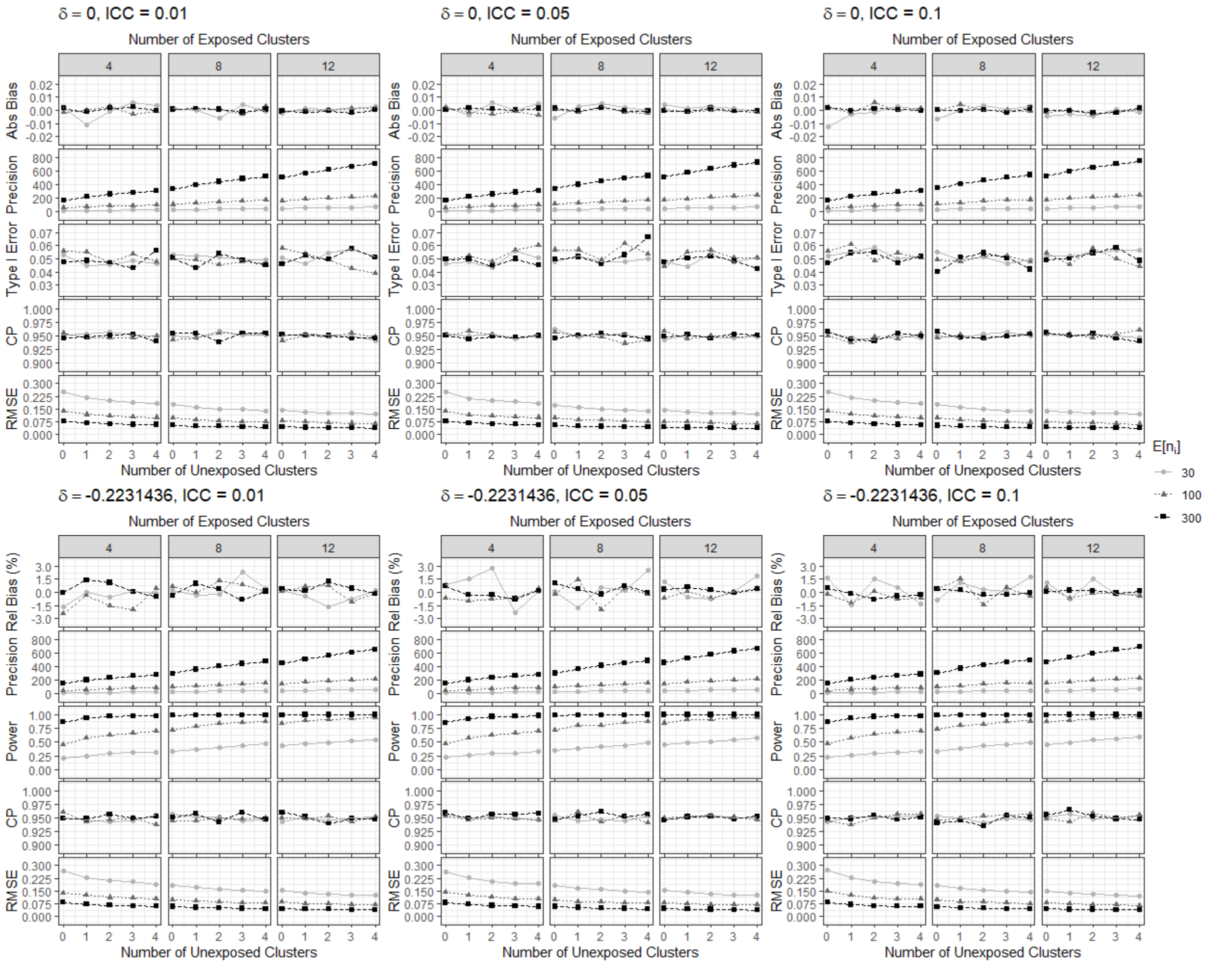

Online Supplementary Material S6. Monte Carlo Standard Errors across different Simulation Scenarios. Monte-Carlo standard errors (standard deviation of the 2,000 estimated intervention effects ($\hat{\delta}_s$) for each scenario) with $\delta = \ln(1.25)$ for binary outcomes and $\delta = \ln(0.80)$ for count outcomes.

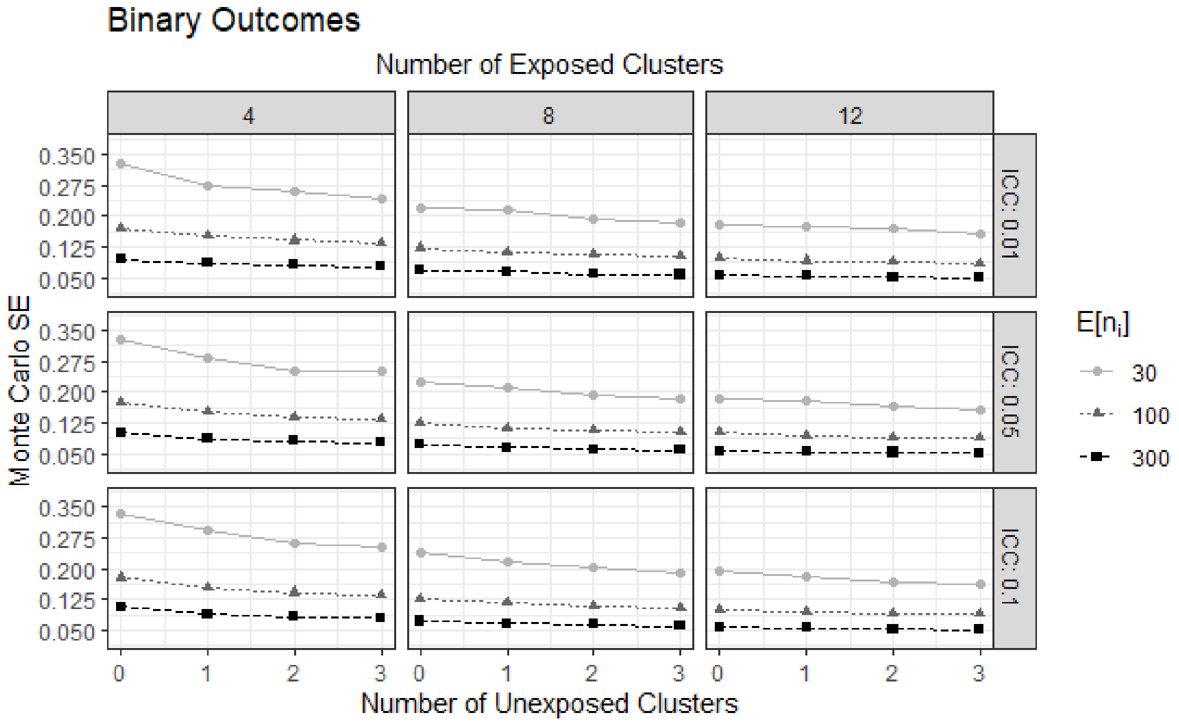

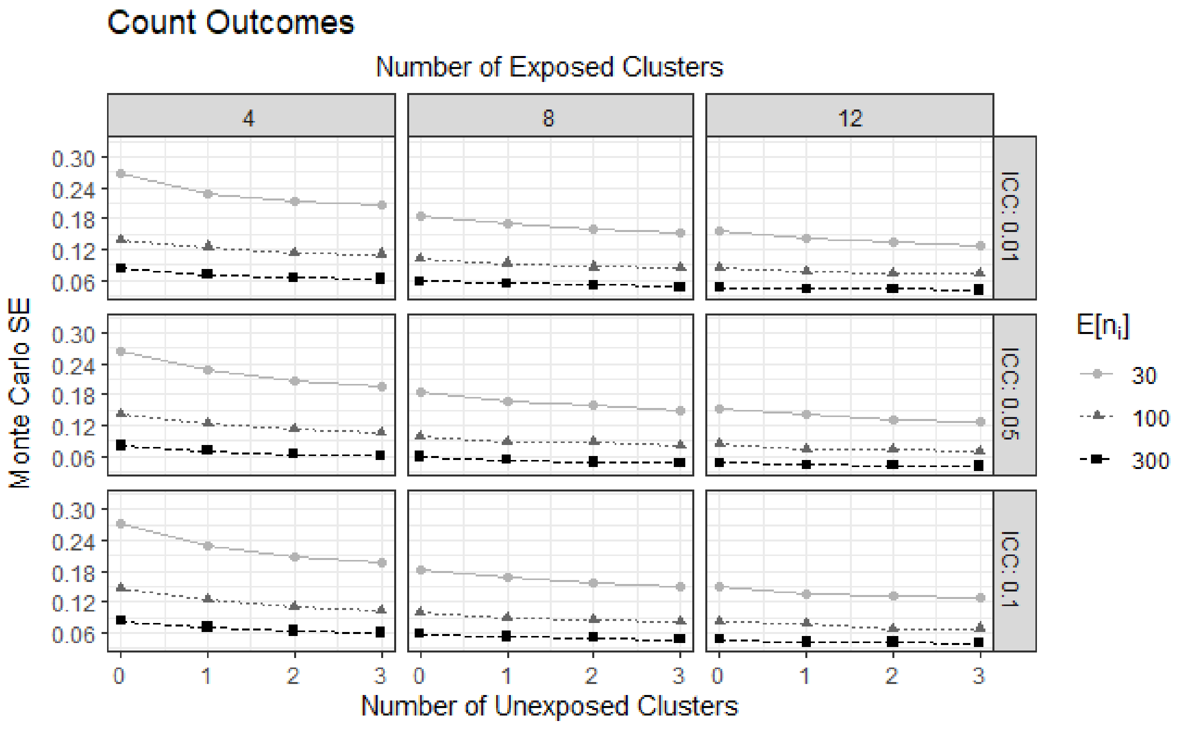

Supplement: Supplementary file 1 — Additional File 1. Online Supplementary Materials S1 to S6 [file 12874_2024_2379_MOESM1_ESM.pdf]
